# Development and External Validation of Individualized Prediction Models for Pain Intensity Outcomes in Patients With Neck Pain, Low Back Pain, or Both in Primary Care Settings

**DOI:** 10.1093/ptj/pzad128

**Published:** 2023-09-26

**Authors:** Lucinda Archer, Kym I E Snell, Siobhán Stynes, Iben Axén, Kate M Dunn, Nadine E Foster, Gwenllian Wynne-Jones, Daniëlle A van der Windt, Jonathan C Hill

**Affiliations:** School of Medicine, Keele University, Keele, Staffordshire, UK; Institute for Applied Health Research, University of Birmingham, Birmingham, UK; National Institute for Health and Care Research (NIHR) Birmingham Biomedical Research Centre, UK; School of Medicine, Keele University, Keele, Staffordshire, UK; Institute for Applied Health Research, University of Birmingham, Birmingham, UK; National Institute for Health and Care Research (NIHR) Birmingham Biomedical Research Centre, UK; School of Medicine, Keele University, Keele, Staffordshire, UK; Midlands Partnership Foundation NHS Trust, North Staffordshire Musculoskeletal Interface Service, Haywood Hospital, Staffordshire, UK; Unit of Intervention and Implementation Research for Worker Health, Institute of Environmental Medicine, Karolinska Institutet, Nobels väg 13, Stockholm, Sweden; School of Medicine, Keele University, Keele, Staffordshire, UK; School of Medicine, Keele University, Keele, Staffordshire, UK; Surgical Treatment and Rehabilitation Service (STARS) Education and Research Alliance, The University of Queensland and Metro North Hospital and Health Service, Queensland, Australia; School of Medicine, Keele University, Keele, Staffordshire, UK; School of Medicine, Keele University, Keele, Staffordshire, UK; School of Medicine, Keele University, Keele, Staffordshire, UK

**Keywords:** Back Pain, External Validation, Neck Pain, Prediction Model Development, Targeted Interventions

## Abstract

**Objective:**

The purpose of this study was to develop and externally validate multivariable prediction models for future pain intensity outcomes to inform targeted interventions for patients with neck or low back pain in primary care settings.

**Methods:**

Model development data were obtained from a group of 679 adults with neck or low back pain who consulted a participating United Kingdom general practice. Predictors included self-report items regarding pain severity and impact from the STarT MSK Tool. Pain intensity at 2 and 6 months was modeled separately for continuous and dichotomized outcomes using linear and logistic regression, respectively. External validation of all models was conducted in a separate group of 586 patients recruited from a similar population with patients’ predictor information collected both at point of consultation and 2 to 4 weeks later using self-report questionnaires. Calibration and discrimination of the models were assessed separately using STarT MSK Tool data from both time points to assess differences in predictive performance.

**Results:**

Pain intensity and patients reporting their condition would last a long time contributed most to predictions of future pain intensity conditional on other variables. On external validation, models were reasonably well calibrated on average when using tool measurements taken 2 to 4 weeks after consultation (calibration slope = 0.848 [95% CI = 0.767 to 0.928] for 2-month pain intensity score), but performance was poor using point-of-consultation tool data (calibration slope for 2-month pain intensity score of 0.650 [95% CI = 0.549 to 0.750]).

**Conclusion:**

Model predictive accuracy was good when predictors were measured 2 to 4 weeks after primary care consultation, but poor when measured at the point of consultation. Future research will explore whether additional, nonmodifiable predictors improve point-of-consultation predictive performance.

**Impact:**

External validation demonstrated that these individualized prediction models were not sufficiently accurate to recommend their use in clinical practice. Further research is required to improve performance through inclusion of additional nonmodifiable risk factors.

## Introduction

Despite substantial research focused on improving patient outcomes in those with neck and/or low back pain (NLBP), the impact of these conditions persists and they are among the top 10 reasons for overall disease burden in terms of disability-adjusted-life-years.^1^ Although most NLBP episodes are not long-lasting, the proportion of people for whom these symptoms develop into disabling problems is growing.^2^ The task of improving first contact treatment for such a large public health problem is an international priority,^3^ particularly among low-income and middle-income countries where the disease burden is rising fastest.^4^

This research builds on epidemiological studies, which have consistently highlighted that the transition from acute NLBP episodes into persistent NLBP can be predicted.^5^^,^^6^ In addition, the use of risk stratification tools to discriminate between risk subgroups to better match initial treatment^7^^,^^8^ has demonstrated advantages to first contact treatment decision-making^3^^,^^9^ and is, therefore, recommended by international guidelines.^10–12^

A key next step is not only to stratify individuals into subgroups based on prognostic information, but to also develop and validate individual patient prediction models and to produce communication aids for consultations, including clear visualizations of predictions. Accurately predicting an individual’s future pain intensity scores may allow for the development of clinician decision support tools that enable more tailored, individualized clinical care. Existing tools, such as the Keele STarT Back risk stratification tool,^8^ determine risk subgroups but do not predict an individual patient’s future pain intensity outcomes, which could be used to shape patient and clinician expectations and lead to more personalized health care.

Existing prediction tools for patients with NLBP have been developed using data collected through self-report questionnaires or interviews, often after first presentation in primary care, rather than during the consultation where most of these tools are intended to be used.^7^^,^^13^^,^^14^ It is important to understand to what extent predictions based on such data align with predictions based on information collected by clinicians during a routine consultation.

This research forms part of a larger body of work, developing digital health technology for first contact consultations to support clinical decision-making for patients with NLBP based on individual outcome predictions, as part of a Horizon 2020 European research program (http://backup-project.eu/).^15^ This incorporates the Keele STarT MSK Tool (www.keele.ac.uk/startmsk), which predicts poor outcomes in patients in primary care settings who are consulting due to musculoskeletal pain,^16^ alongside a set of recommended risk-matched treatment options developed through consensus.^9^^,^^17^^,^^18^

In the present study, we used predictor items that were agreed to be clinically relevant during the forming of the Keele STarT MSK Tool to develop new models to predict an individual’s future pain intensity. We report on the development, internal validation, and external validation of prognostic models to predict 2- and 6- month pain intensity score, which was also modeled when dichotomized as low/moderate-high pain intensity. We explored the predictive performance of these models in external data, using predictor information collected at 2 distinct time points: during the consultation and through patient self-report questionnaires collected 2 to 4 weeks afterwards.

## Methods

### Source of Data

For this study, secondary analysis of data from 2 existing datasets, the Keele Aches and Pains Study (KAPS)^16^ and the STarT MSK Pilot Trial (STarT MSK-pilot),^9^ was combined for model development and internal validation, while external validation of the prediction models was conducted in patients from a third existing dataset: the STarT MSK Main Trial (STarT MSK-MT).^18^ Eligible patients were defined in the same way in all 3 datasets: those aged 18 and over, consulting at a participating general practice with musculoskeletal pain. The present study included only the subset of patients who consulted with NLBP. Further details of the datasets used in these analyses are included in Supplementary Appendix I.

### Outcome Definitions

The outcome was future pain intensity score, which was measured at 2- and 6-month follow-up, through participants’ self-reported response to the question “*How intense was your pain, on average, over the last 2 weeks? [Responses on a 0-10 scale, where 0 is* ‘*no pain*’ *and 10 is* ‘*worst pain ever*’].” This score was modeled continuously and separately as a binary outcome, dichotomized as 0 to 4 (low pain intensity) versus 5 to 10 (moderate-high pain intensity). A cut-off of 5 on a 0–10 numerical rating scale to indicate at least moderate pain intensity has been reported previously in the literature^19–21^ and was considered clinically meaningful by the physical therapists in the research team (ie, was considered to be the most appropriate cut-point to group patients into those with good pain intensity outcomes [score of 0 to 4] and those with poor pain intensity outcomes [5 or more]).

### Predictors

The 10 items from the Keele STarT MSK Tool were considered as predictors in all models (Tab. 1). These were pain intensity (on a scale from 0 to 10), pain self-efficacy, pain impact, walking short distances only, pain elsewhere, thinking their condition will last a long time, other important health problems, emotional well-being, fear of pain-related movement, and pain duration.^16^ No statistical selection was conducted, as these predictor variables had all been considered clinically important during the development of the Keele STarT MSK Tool.^16^ We included the additional predictor of primary pain site (back or neck pain) as this was identified through discussion with the wider research team as being potentially clinically important for both accurate prediction and face validity.

**Table 1:** Baseline Predictor Responses*^a^*

	**Model Development**			**External Validation**	
	**STarT MSK-Pilot**	**KAPS**	**Total**	**POC**	**2–4 Wk**
	*n =* 214	*n* = 465	*n =* 679	*n =* 275	*n* = 586
Age (at consultation, y), mean (SD)	58.2 (15.9)	54.6 (17)	55.7 (16.7)	56.6 (15.5)	58.5 (16.0)
Sex, female	134 (62.6)	267 (57.4)	401 (59.1)	167 (60.7)	353 (60.2)
Primary pain site, neck	59 (27.6)	57 (12.3)	116 (17.1)	61 (22.2)	129 (22.0)
Pain duration					
<3 mo	67 (31.3)	116 (25.0)	183 (27.0)	63 (22.9)	140 (23.9)
3–6 mo	33 (15.4)	67 (14.4)	100 (14.7)	41 (14.9)	91 (15.5)
7–12 mo	29 (13.6)	33 (7.1)	62 (9.1)	33 (12.0)	70 (12.0)
Over 1 y	85 (39.7)	240 (51.6)	325 (47.9)	134 (48.7)	278 (47.4)
Comorbidities (self-reported)					
Diabetes	19 (8.9)	41 (8.8)	60 (8.8)	23 (8.4)	59 (10.0)
Respiratory problem	37 (17.3)	67 (14.4)	104 (15.3)	48 (17.5)	102 (17.4)
Heart problem	65 (30.4)	118 (25.4)	183 (27.0)	75 (27.3)	171 (29.2)
Chronic fatigue	14 (6.5)	11 (2.4)	25 (3.7)	16 (5.8)	43 (7.3)
Anxiety/depression	48 (22.4)	97 (20.9)	145 (21.4)	61 (22.2)	147 (25.1)
Other	46 (21.5)	121 (26.0)	167 (24.6)	71 (25.8)	132 (22.5)
EQ5D—Usual activities					
No problem	44 (20.6)	138 (29.7)	182 (26.8)	40 (14.6)	96 (16.4)
Slight problem	80 (37.4)	139 (29.9)	219 (32.3)	95 (34.6)	206 (35.2)
Moderate problem	52 (24.3)	106 (22.8)	158 (23.3)	86 (31.3)	183 (31.2)
Severe problem	28 (13.1)	60 (12.9)	88 (13.0)	38 (13.8)	70 (12.0)
Unable/extreme problem	10 (4.7)	15 (3.2)	25 (3.7)	12 (4.6)	23 (3.9)
Help to read instructions					
Never	162 (75.7)	354 (76.1)	516 (76.0)	221 (80.4)	476 (82.2)
Rarely	33 (15.4)	48 (10.3)	81 (11.9)	22 (8.0)	51 (8.7)
Sometimes	9 (4.2)	36 (7.7)	45 (6.6)	19 (6.9)	32 (5.5)
Often	2 (0.9)	14 (3.0)	16 (2.4)	9 (3.3)	19 (3.2)
Always	3 (1.4)	13 (2.8)	16 (2.4)	1 (0.4)	2 (0.3)
NDI—Baseline score, mean (SD)	16.1 (8)			17.5 (8.7)	16.6 (8.7)
NDI %—Baseline score, mean (SD)	32.2 (16)			35.1 (17.4)	33.2 (17.4)
RMDQ—Baseline score, median (IQR)	9 (5–13)			10 (5–15)	9 (5–14)
STarT MSK Tool score (baseline), median (IQR)	6 (4–7)	7 (4–9)	7 (4–9)	7 (5–8)	7 (5–9)
STarT MSK Tool score subgroup (baseline)					
High risk	29 (13.6)	118 (25.4)	147 (21.6)	95 (34.6)	140 (23.9)
Medium risk	105 (49.1)	176 (37.9)	281 (41.4)	120 (43.6)	273 (46.6)
Low risk	63 (29.4)	125 (26.9)	188 (27.7)	40 (14.6)	101 (17.4)
1) Pain intensity, median (IQR) *On average, how intense was your pain* [*where 0 is* “*no pain*” *and 10 is* “*pain as bad as it could be*”]?	7 (5–8)	7 (4–8)	7 (5–8)	7 (6–8)	7 (5–8)
2) Pain self-efficacy *Do you often feel unsure about how to manage your pain condition?*	144 (67.3)	225 (48.4)	369 (54.3)	163 (59.3)	279 (47.6)
3) Pain impact *Over the last 2 wk, have you been bothered a lot by your pain?*	113 (52.8)	330 (71.0)	443 (65.2)	228 (82.9)	462 (78.8)
4) Walking short distances only *Have you only been able to walk short distances because of your pain?*	117 (54.7)	248 (53.3)	365 (53.8)	143 (52.0)	344 (58.7)
5) Pain elsewhere *Have you had troublesome joint or muscle pain in more than 1 part of your body?*	147 (68.7)	304 (65.4)	451 (66.4)	126 (45.8)	398 (67.9)
6) Thinking their condition will last a long time *Do you think your condition will last a long time?*	154 (72.0)	315 (67.7)	469 (69.1)	211 (76.7)	477 (81.4)
7) Other important health problems *Do you have other important health problems?*	81 (37.9)	183 (39.4)	264 (38.9)	88 (32.0)	242 (41.3)
8) Emotional well-being *Has pain made you feel down or depressed in the last 2 wk?*	132 (61.7)	284 (61.1)	416 (61.3)	170 (61.8)	388 (66.2)
9) Fear of pain-related movement *Do you feel it is unsafe for a person with a condition like yours to be physically active?*	56 (26.2)	133 (28.6)	189 (27.8)	144 (52.4)	322 (55.0)
10) Pain duration *Have you had your current pain problem for 6 months or more?*	114 (53.3)	219 (47.1)	333 (49.0)	121 (44.0)	345 (58.9)

^*a*^Baseline characteristics and responses to the Keele STarT MSK tool items (“As you answer these questions, think about how you have been over the last two weeks:”) in model development and external validation data sets. Values are *n* (%) unless otherwise stated. EQ5D = EuroQol 5 Dimension; IQR = interquartile range; KAPS = Keele Aches and Pains Study; NDI = Neck Disability Index; POC = point of consultation; RMDQ = Roland Morris Disability Questionnaire; SD = standard deviation.

Predictor information was collected through a postal questionnaire sent to patients within a few days of their general practice consultation (KAPS, STarT MSK-pilot, and STarT MSK-MT). For STarT MSK-MT (the external validation data), predictor information was also collected at the time of general practice consultation in patients in the intervention arm, and these data were used for additional validation analyses.

Further detail on all candidate predictors is given in Supplementary Appendix IV (Tab. S1).

### Statistical Analysis

#### Sample Size

**Figure 1:**
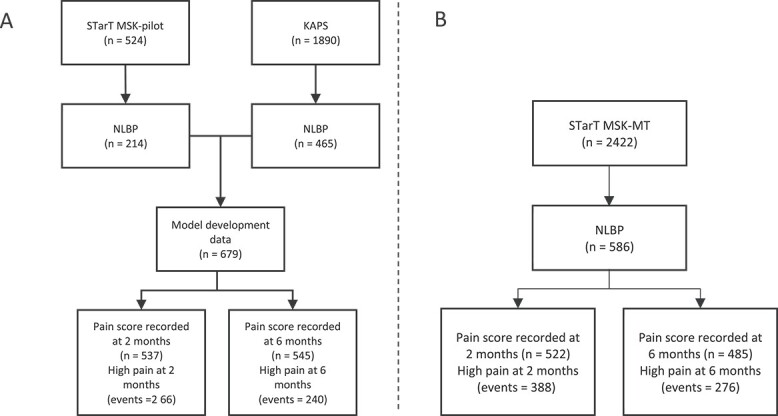
Patient flow summary at model development (A) and at external validation (B). KAPS = Keele Aches and Pains Study; NLBP = neck and/or low back pain.

The sample size for all analyses was fixed due to the size of the available datasets. We compared the available number of participants for each analysis (Fig. 1) to sample size recommendations for developing^22^^,^^23^ and externally validating^24–26^ clinical prediction models. Further details on the sample size calculations are included in Supplementary Appendix II.

Based on the anticipated inclusion of 11 predefined predictor parameters (1 continuous predictor, modeled linearly, and 10 binary predictors), we required 311 participants for the development of models for continuous pain intensity score, and at least 824 participants (with 412 “moderate-high pain intensity” events) for binary pain intensity outcomes. Thus, our available data exceeded the requirements for the continuous pain intensity score model but was not enough for the binary pain intensity outcome.

For precise estimation of model performance, we required 892 and 1946 participants to externally validate the continuous and binary outcome models, respectively; thus, estimates of predictive performance are subject to some uncertainty.

#### Missing Data

Multiple imputation by chained equations was used to account for missing data in both predictor and outcome measurements, under the assumption that data were missing at random.^27^^,^^28^ Multiple imputation was performed separately for each dataset to allow for the clustering of individuals within that dataset, with the number of imputations chosen to exceed the percentage of incomplete cases.^28^ Preliminary checks for associations between missingness and predictor values were conducted to check for obvious violations of the missing at random assumption. Results of analyses were pooled across imputations using Rubin rules where appropriate.^27^

#### Model Development

Continuous pain intensity score outcomes were modeled using random-effects linear regression, while binary outcomes of moderate-high pain intensity were modeled using random-effects logistic regression.^29^ Outcomes were modeled using multilevel mixed-effects models to account for heterogeneity across the 2 model development datasets, resulting in average model intercepts across the KAPS and STarT MSK-pilot datasets.^30^ Models were fitted using restricted maximum likelihood (REML), with an unstructured variance–covariance for the random effects on the intercept term.^31^ Continuous predictors were modeled linearly on their continuous scale, and all predictors were forced into all models.^32^^,^^33^

#### Internal Validation

Predictive performance of the developed models was assessed through calibration for the continuous outcome models, and through calibration and discrimination for the binary outcome models.^34^ Calibration was assessed using the calibration slope, calibration in the- large (CITL), and the ratio of observed to expected cases (O/E, for binary outcome models only). Discrimination was assessed through the C statistic. The proportion of variance in the outcome explained by the predictors in each model was determined using the adjusted ${R}^2$ (or pseudo ${R}^2$ for binary outcomes, using Nagelkerke and Cox-Snell approaches).

Internal validation was conducted simultaneously for all models, using bootstrapping with 1000 samples to provide optimism-adjusted estimates of predictive performance.^27^^,^^29^^,^^35^

The optimism-adjusted calibration slope was used as an estimate of the uniform shrinkage factor for each model, with regression coefficients multiplied by this shrinkage factor to correct for overfitting.^29^^,^^35^^,^^36^

#### External Validation

Model equations for the 4 prediction models were applied for the participants in the STarT MSK-MT data to calculate the prediction values from each model. Predictive performance measures were calculated, as described for the internal validation, including measures of calibration (calibration slope, CITL, O/E ratio) and discrimination (C statistic), and measures of overall model fit (${R}^2$ or Nagelkerke pseudo ${R}^2$ for binary outcomes). Model performance was also assessed within subgroups to check consistency in performance across age ranges, sex, treatment group (matched treatment or usual care), and pain durations prior to presentation.

Predictors in the STarT MSK-MT data were recorded for each patient at 2 time points. Data on predictors were available for each participant when assessed (i) within the general practice consultation, and (ii) after the consultation using a self-reported questionnaire, which was returned by post around 2 to 4 weeks after the consultation.

The timing of risk predictors collected 2 to 4 weeks after consultation was more similar to the recording of the predictor variables in the model development data, while predictor variable collection at the point of consultation better reflects the models’ intended future use. We therefore tested model performance for both data collection time points to assess the validity of our assumption that the developed models could be used in practice at point of consultation.

**Table 2:** Prognostic Models*^a^*

	Coefficients for Continuous Outcome, Pain Score (β)		Odds Ratios for Binary Outcome, High Pain	
	2 mo	6 mo	2 mo	6 mo
Pain intensity	0.236	0.269	1.23	1.26
Pain self-efficacy	0.526	0.212	1.38	1.20
Pain impact	0.859	0.632	2.33	1.40
Walking short distances only	0.447	0.934	1.57	2.37
Pain elsewhere	0.49	0.278	1.51	1.33
Thinking their condition will last a long time	1.22	1.673	2.51	3.61
Other important health problems	0.783	0.578	1.73	1.38
Emotional well-being	−0.032	−0.002	0.91	1.28
Fear of pain-related movement	0.041	−0.434	1.07	0.63
Pain duration	1.029	1.129	2.82	2.19
Primary pain site	−0.171	0.515	0.69	1.02
Intercept	−0.304	−1.153	−4.010	−4.324
Var (intercept)	0.237	0.132	0.030	0.041
Shrinkage factor	0.975	0.982	0.930	0.938

^*a*^Prognostic models after optimism adjustment. Numbers are intercepts (α) and coefficients (β) and for continuous outcome models, intercepts (α), and odds ratios (exp[β]) and for binary outcome models. Uniform shrinkage factors for each model were obtained through bootstrapping with 1000 replications.

Extended statistical methods are given in Supplementary Appendix III. All analyses were performed using Stata MP Version 16 (StataCorp). This paper adheres to the TRIPOD checklist for the transparent reporting of multivariable prediction models, see Supplementary Appendix VI.^37^

### Role of the Funding Source

The funders played no role in the design, conduct, or reporting of this study.

## Results

### Study Population

#### Development Data

Across the 2 model development datasets, 679 patients with NLBP were available for inclusion in the analysis (Fig. 1A). Patients predominantly presented with back pain (83%), with a much smaller group presenting with neck pain (17%), and had a median baseline pain intensity score of 7 (interquartile range = 5–8) out of 10. Patient demographics across the 2 datasets are given in Table 1.

Table 1 also shows a summary of predictor responses. When summarized across both model development datasets, most patients (*n* = 451, 66.4%) had troublesome musculoskeletal pain in more than 1 part of their bodies, with 69.1% (*n* = 469) thinking their condition would last a long time. A further 27.8% (*n* = 189) participants were reported a “fear of pain-related movement.”

Few predictors’ variables showed substantial differences (larger than 10%) in distribution between the STarT MSK-pilot and KAPS datasets, as can be seen in Table 1. Notable differences in predictor variable distributions included those for pain self-efficacy (“*unsure about how to manage* [their] *pain condition*”; STarT MSK-pilot 67.3%, KAPS 48.4%); and for pain impact (“*bothered a lot by* [their] *pain*” in the preceding 2 weeks; STarT MSK-pilot 52.8%, KAPS 71.0%).

#### External Validation Data

The STarT MSK-MT data included 586 patients with NLBP for the external validation of the above models (Fig. 1B). Patients predominantly presented with back pain (78%) and had a median baseline pain intensity score of 7 (Interquartile range [IQR] = 6–8) out of 10 reported at point of consultation, and a median of 7 (IQR = 5–8) when reporting in the data collected 2 to 4 weeks after consultation.

The majority of patients reported experiencing moderate-high pain intensity at 2 months, with a prevalence of moderate-high pain intensity at 57.7% (slightly higher than the 49.5% prevalence seen in the development data). This dropped to 47.1% at 6 months follow-up (44.0% for the development data). Baseline pain intensity scores on a scale from 0 to 10 were reported consistently across the data collected 2 to 4 weeks after consultation (median = 7; IQR = 5–8) and at the point of consultation (median = 7; IQR = 6–8), with a slightly narrower spread of scores recorded at point of consultation.

**Figure 2:**
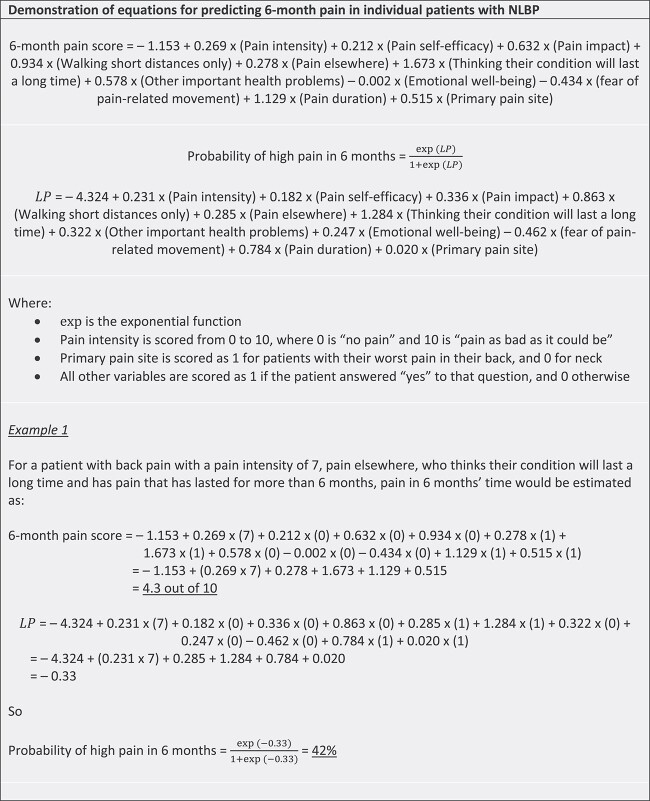
Demonstration of prediction calculation. LP = linear predictor; NLBP = neck and/or low back pain.

**Table 3:** External Validation, Predictive Performance *^a^*

Time	Outcome	Measure	Point of Consultation	2–4 Wk After Consultation
				
2 mo	Pain score	Calibration slope	0.650 (0.549 to 0.750)	0.848 (0.767 to 0.928)
		CITL	0.649 (0.506 to 0.792)	0.378 (0.249 to 0.507)
		*R* ^2^ median (IQR)	11.1% (10.0% to 12.1%)	25.3% (24.9% to 26.0%)
				
	High pain	Calibration slope	0.436 (0.338 to 0.535)	0.657 (0.556 to 0.758)
		CITL	1.081 (0.951 to 1.21)	0.798 (0.665 to 0.931)
		O/E	1.599 (1.552 to 1.647)	1.369 (1.329 to 1.41)
		C statistic	0.649 (0.618 to 0.679)	0.726 (0.696 to 0.753)
		Pseudo *R*^2^ median (IQR)	12.1% (10.5% to 13.3%)	27.1% (26.1% to 27.9%)
				
6 mo	Pain score	Calibration slope	0.593 (0.499 to 0.688)	0.735 (0.656 to 0.815)
		CITL	−0.93 (−1.088 to −0.773)	−1.262 (−1.408 to −1.116)
		*R* ^2^ median (IQR)	10.4% (9.4% to 11.4%)	20.8% (20.2% to 21.3%)
				
	High pain	Calibration slope	0.526 (0.417 to 0.635)	0.71 (0.598 to 0.823)
		CITL	0.028 (−0.101 to 0.157)	−0.307 (−0.438 to −0.176)
		O/E	1.015 (0.984 to 1.046)	0.88 (0.854 to 0.908)
		C statistic	0.663 (0.632 to 0.693)	0.721 (0.692 to 0.749)
		Pseudo *R*^2^ median (IQR)	8.7% (7.6% to 10.2%)	22.1% (21.1% to 23.1%)
				

^*a*^Predictive performance of models for pain on external validation in the STarT MSK Main Trial data. CITL = calibration in the large; IQR = interquartile range; O/E = observed/expected ratio.

Predictors regarding pain impact and pain self-efficacy were both reported as present in a higher proportion of patients when collected at point of consultation, while the remaining predictor items showed a higher prevalence when recorded 2 to 4 weeks after consultation. The largest difference was seen for the “pain elsewhere” item, with 68% answering “yes” 2 to 4 weeks after consultation compared to only 46% answering “yes” at point of consultation.

### Model Development and Internal Validation

The final models for predicting pain intensity scores in patients with NLBP, after optimism adjustment, are presented in Table 2, along with the shrinkage factor estimated via bootstrapping. Detailed results from the internal validation can be seen in Supplementary Appendix IV (Tab. S2 and Fig. S1).

Conditional on other variables in the model, baseline pain intensity and thinking their condition would last a long time were the strongest predictors of pain intensity at both follow-up time points, with higher baseline pain intensity and expecting the condition to last a long time both being associated with higher pain intensity scores at follow-up. Episode duration (whether the patient had experienced pain for longer than 6 months at the time of their general practice consultation) was also an important predictor, associated with higher pain intensity at both 2 and 6 months. Figure 2 gives a demonstration of how the models for pain intensity could be used to calculate predictions for pain intensity score and the probability of moderate-high pain intensity for individual patients at 6 months.

### External Validation

**Figure 3:**
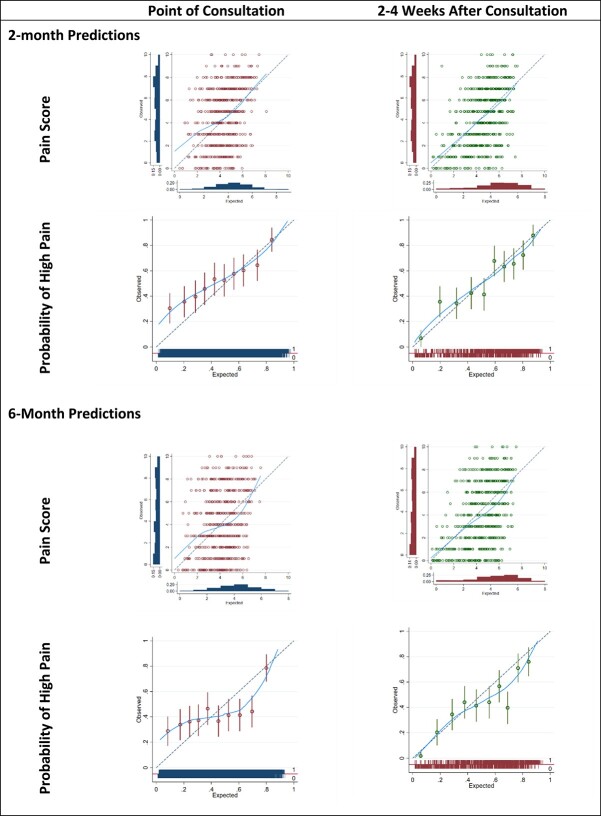
Model calibration in external validation data.

Details of the model performance on external validation are given in Table 3, while calibration plots for all models can be seen in Figure 3.

Predictions for continuous pain intensity score generated using point-of-consultation data were poorly calibrated when compared to observed values, with calibration slopes of 0.65 (0.55–0.75) and 0.59 (0.50–0.69) at 2 and 6 months respectively. However, the predictions generated in data from 2 to 4 weeks after consultation showed better calibration at both time points, with calibration slopes of 0.85 (0.77–0.93) and 0.74 (0.66–0.82).

Estimates of CITL suggest that the 2-month pain intensity score model systematically underpredicted patients 2-month pain intensity scores by an average of 0.65 pain intensity points in the point-of-consultation predictions (95% CI = 0.51 to 0.79), and by an average of 0.38 pain intensity points (95% CI = 0.25 to 0.51) in predictions generated from predictor responses 2 to 4 weeks after consultation. The pain intensity score model at 6 months systematically overpredicted patients’ pain intensity scores for both sources of predictor data, with predicted pain intensity scores being an average of 0.93 points too high in the point-of-consultation data (95% CI = 0.77 to 1.09), and 1.20 points too high in the data from 2 to 4 weeks after consultation (95% CI = 1.12 to 1.41).

The model to predict pain intensity score at 2 months performed better than the 6-month model by all predictive performance measures, in both sources of predictor variables at point of consultation and 2 to 4 weeks after consultation, as can be seen in Table 3.

Calibration performance was poor for the models predicting the binary outcome of moderate-high pain intensity. In the point-of-consultation data, the calibration slope was 0.44 (95% CI = 0.34 to 0.54) for predicting high pain intensity at 2 months and 0.53 (95% CI = 0.42 to 0.64) at 6 months, indicating predictions were too high in those at low risk of high pain intensity and were too low in those at high risk of moderate-high pain. As with the continuous outcome pain intensity models, calibration slopes indicated better calibration performance for predictions generated using predictors collected 2 to 4 weeks after consultation.

Discrimination performance was consistent for models predicting moderate-high pain intensity at both time points. C statistics of 0.65 (0.62–0.68) and 0.66 (0.63–0.69) at 2 and 6 months, respectively, suggest that around 65% and 66% of concordant pairs were correctly identified by the models for these outcome time points, based on predictors recorded at point of consultation. Again, using predictor values from 2 to 4 weeks after consultation to generate predictions gave better discriminative performance, with 73% of concordant pairs correctly identified at 2 months and 72% identified at 6 months.

Analyses to assess model performance across different subgroups (shown in Suppl. Appendix V) suggest that model performance was reasonably consistent across age ranges, sex, treatment group, and pain duration prior to presentation.

## Discussion

We have developed and externally validated new individualized prediction models for pain intensity outcomes at 2- and 6-month follow-up in patients consulting with NLBP in general practice, based on the Keele STarT MSK Tool items.

The findings from our external validation demonstrated that models applied in the predictor data collected 2 to 4 weeks after the consultation achieved better predictive performance than data collected at the point of consultation. For example, in terms of calibration performance, the model predicting pain intensity score at 2 months had a calibration slope of 0.848 (0.767–0.928) in data from 2 to 4 weeks after consultation, but only 0.650 (0.549–0.750) at point of consultation. The discriminative ability of the models for predicting 6-month binary outcomes was more consistent between validations in data from 2 to 4 weeks after consultation and point of consultation, with a reasonable C statistic of 0.66 for moderate–high pain intensity when using data collected at point of consultation compared with 0.72 for predictions based on data from 2 to 4 weeks after consultation. The continuous outcome models showed a systematic underprediction of pain intensity scores at 2 month, and systematic overprediction at 6 months. When predictions were generated using data collected at point of consultation, this overprediction was by around 0.9 points on the 0–10 pain intensity scale, which is not trivial.

The models presented here were based on known predictors for outcomes in patients with NLBP from the team’s previous risk stratification work.^16^ The choice of candidate predictors was therefore limited to our previously validated Keele STarT MSK Tool items: A decision that proved in hindsight to be a limitation to exploring options for greater accuracy in our predictions of pain intensity outcomes. In understanding reasons for the disappointing performance of these prediction models, it is important to recognize that the Keele STarT MSK Tool was designed for risk-stratification to inform clinicians about risk-matched treatment options. The Keele STarT MSK Tool, therefore, contains mainly items considered to be treatment modifiable risk factors (with pain duration being the only nonmodifiable item). Other known non-modifiable risk factors that were not considered during the model development process presented here include factors such as employment status and other socioeconomic indicators, previous surgery, comorbidities, and previous pain episodes.^38^ The next step to improve model performance for individualized predictions will be to explore whether adding such nonmodifiable factors to our models improves their predictive accuracy.

### Comparison With Other Studies

Other prediction models currently exist to predict various outcomes in those with NLBP, such as time to recovery for patients with acute low back pain,^13^ global perceived effect for patients with persistent neck pain,^39^ or disability in patients undergoing surgery for lumbar degeneration.^40^ However, as far as we are aware, this is the first time that models have specifically been developed to predict an individual patient’s levels of pain intensity at future time points. Prediction models in the field which have previously been updated and externally validated^13^^,^^41^ often performed suboptimally in external samples. It is not uncommon for risk prediction models to need updating depending on their specific purpose or clinical population, as we have found here.

Several studies have previously shown baseline pain intensity to be a reliable predictor of future pain intensity among patients with low back pain, suggesting that initial pain levels could be a useful indicator of long-term pain outcomes and poor recovery.^42–44^ Our findings are also consistent with recent data from Brazil showing that the Keele STarT Back Tool is more predictive of outcomes when collected a few weeks after a physical therapist consultation than at the consultation itself.^28^ Our results further agree with a study in United Kingdom and Dutch primary care which suggests that additional assessment of pain intensity after 4 to 6 weeks results in better predictions than when using baseline pain intensity alone.^45^ However, although predictor–outcome associations are known to be weaker for time-varying predictors (such as pain intensity) when measured at consultation than when measured 2 to 4 weeks after consultation, it is still recommended for such predictors to be measured at the time of intended model use to improve the applicability of the model in practice.^46^

In patients with neck pain, expectations and previous clinical course of symptoms were found to predict global perceived effect.^41^ In patients with low back pain of short duration (<4 weeks) and a pain intensity ≥2/10, the duration of the current episode, pain intensity, and depression was found to predict time to recovery from pain.^14^ Despite some differences in populations, we also found that pain intensity, episode duration, and thinking their condition will last a long time to predict future pain intensity (conditional on other model variables). Thus, some predictive factors seem consistent across the literature.

Our findings of the limited predictive performance of low mood, however, contrast with the results of an umbrella review of systematic reviews in this area.^38^ Differences in the predictive ability of mood may be related to differences in the populations used for analysis, or due to other variables in our models (such as bothersomeness or pain intensity) assimilating the prognostic impact of mood.

It should be noted that all variables included in our models were self-report measures, with no variables arising from clinical examination, existing electronic medical record data, or imaging results. This decision was made due to a lack of a standardized clinical examination for patients consulting with NLBP, and the expectation that using self-report items could overcome the wide variation in general practitioner clinical examinations. Furthermore, general practitioners rarely have imaging results on which to base a treatment decision. Indeed, previous work suggests that clinical examination and MRI scan results add little to outcome predictions in patients with low back pain over-and-above predictors such as younger age, attitudes and beliefs regarding pain, or depression.^47^^,^^48^

### Strengths and Limitations

We acknowledge that to achieve a comprehensive understanding of a patient’s health status over time, a variety of outcomes are needed. Therefore, a key limitation here is that our new prognostic model focusses solely on predicting pain intensity. Although pain intensity is undoubtedly an essential aspect of measuring a patient’s pain experience, it does not reflect the complexity of pain and its wider impact on an individual’s physical, emotional, and social well-being. We are, therefore, planning to similarly publish prognostic models for a broader range of outcomes, including physical function (restriction in usual activities) and time off work. Collectively, these models will provide useful information that could inform treatment decisions and guide patient care.

A barrier to implementation in practice is the complexity for clinicians to calculate outcome predictions for individual patients, which may require a prebuilt calculator. Such a calculator has been incorporated into the Back-UP first contact WebApp dashboard,^15^ however, reflecting on the relatively low Cstatistic seen within the external validation at the point of consultation; however, further research is needed to improve the discriminative performance of these models before we could recommend use in clinical practice.

Evaluating the performance of predictions at the point of consultation is a clear strength to our validation, with predictive performance assessment at the point where the models are intended to be used in practice. However, small sample sizes for this external validation resulted in some uncertainty around performance estimates in this population, where all models performed less well than when used in data from 2 to 4 weeks after consultation. Although further external validation in a larger dataset would reduce our uncertainty in predictive performance estimates, the current validation gives a good indication that for these models to perform well at point of consultation, where they would be used in practice, it is likely that updating (for example, to incorporate nonmodifiable risk factors) or recalibration would be needed.

A strength of the external validation is in the representativeness of the sample used. An anonymized medical record audit of all patients with MSK conditions in primary care settings suggested that there was no evidence of selection bias in baseline pain intensity or risk severity in the participants within the STarT MSK MT population. Therefore, we are confident that the sample used was representative of patients consulting to primary care in the United Kingdom.

It was not possible to produce separate prediction models for patients with neck pain and lower back pain, due to the limited number of patients available with the neck as their primary pain site. Rather than neglecting to include these patients with neck pain in our analyses, we instead combined the patient populations and introduced “primary pain site” as a predictor in the model. For this reason, predictor effects are likely to be highly weighted toward the effects experienced by patients with low back pain as their primary pain site, and thus future validation in separate neck pain and low back pain populations would be required to assess the extent to which our conclusions (and models) can be applied to a population with neck pain only.

### Implications

Predictions of binary pain intensity outcomes, as included here, provide clinicians and patients with a simple assessment of expected pain intensity at future time points. These offer results that are easy to interpret and can contribute quickly to decision making in clinical settings. However, binary pain intensity outcomes do not provide detailed information about the magnitude or severity of pain, which may limit their usefulness for monitoring changes in pain intensity over time. In contrast, pain intensity predictions on the continuous scale, which this study also presents, give a clear indication of the change in pain intensity score over time, allowing identification of clinically meaningful, smaller changes in expected pain intensity. Analyzing pain intensity outcomes on the continuous scale maximizes the available information for detecting predictor–outcome associations and allows these models to have greater flexibility to be used in different contexts or locations, where a different dichotomy of pain intensity score might be preferred.

The initial individualized prediction models developed within this study were incorporated into an online demonstrator, the Back-UP First Contact web app (http://backup-project.eu/?p=767).^15^ For the first time, clinicians were able to see individualized patient predictions on hypothetical patients and give their feedback to the research team about the usability of individual predictions and visualizations to inform treatment decision-making. A future paper will report on the acceptability of the prediction visualizations to both clinicians and patients. The findings of this study, however, suggest that, at present, the prediction models we have are not yet adequate for clinical use for prediction purposes at the point of consultation. Further research is therefore required to improve the prediction models.

### Conclusion

We have developed and externally validated models to predict pain intensity outcomes for individual patients consulting in primary care with NLBP. The variables included within the risk prediction models were limited to the existing Keele STarT MSK Tool items. External validation demonstrated that these individualized prediction models, particularly when evaluated at the point of consultation, were not sufficiently accurate to recommend their use in clinical practice. Further research is therefore required to improve the prediction models through inclusion of additional nonmodifiable risk factors.

## Supplementary Material

2022_0584_r2_si_tsr_pzad128

## Data Availability

The datasets analyzed during the current study are available from the corresponding author on reasonable request.
