# Supplementary material for: Development and External Validation of Individualized Prediction Models for Pain Intensity Outcomes in Patients With Neck Pain, Low Back Pain, or Both in Primary Care Settings
Source: Phys Ther. 2023 Sep 26;103(11):pzad128. doi: 10.1093/ptj/pzad128 (PMC10682973; doi:10.1093/ptj/pzad128)
Supplement: 2022_0584_r2_si_tsr_pzad128 [file 2022_0584_r2_si_tsr_pzad128.pdf]

Supplementary material: Development and external validation of individualised prediction models for pain outcomes in primary care consulters with neck and/or low back pain

Authors

Lucinda Archer[1], Kym IE Snell[1], Siobhán Stynes[1,2], Iben Axen[3], Kate M Dunn[1], Nadine E Foster[1,4], Gwenllian Wynne-Jones[1], Daniëlle A van der Windt[1], Jonathan C Hill[1*]

[1] School of Medicine, Keele University, Staffordshire, ST5 5BG, United Kingdom

[2] Midlands Partnership Foundation NHS Trust, North Staffordshire Musculoskeletal Interface Service, Haywood Hospital, Staffordshire, United Kingdom.

[3] Karolinska Institutet. Institute of Environmental Medicine, Unit of Intervention and Implementation Research for Worker Health. Nobels väg 13, 171 77 Stockholm, Sweden.

[4] Surgical Treatment and Rehabilitation Service (STARS) Education and Research Alliance, The University of Queensland and Metro North Hospital and Health Service, Queensland, Australia.

* corresponding author: Jonathan Hill

Email: j.hill@keele.ac.uk

tel: +44 (0) 1782 733900

Funding

- This paper presents work conducted as part of a project funded by the European Horizon 2020 research and innovation programme under grant agreement No 777090. The authors have no conflicts of interest to declare.
- It uses data collected as part of independent research funded by the National Institute for Health Research (NIHR) under its Programme Grants for Applied Research scheme (grant number: STarT MSK programme RP-PG-1211-20010) as well as Centre of Excellence funding from Versus Arthritis (grant reference: 20202).
- Lucinda Archer and Kym Snell were supported by funding from the Evidence Synthesis Working Group, which was funded by the NIHR School for Primary Care Research (NIHR SPCR) [Project Number 390].
- Kym Snell was funded by the NIHR School for Primary Care Research (NIHR SPCR Launching Fellowship)
- Nadine Foster was an NIHR Senior Investigator, and was supported through an NIHR Research Professorship (NIHR-RP-011-015).
- The funding bodies had no role in the design of the study and collection, analysis, interpretation of data, or in writing the manuscript.
- The views expressed in this article are those of the authors and not necessarily those of the EU, NHS, the NIHR, our funding bodies or the Department of Health and Social Care

Appendix I: Detailed description of data sources

Model development data

i) Keele Aches and Pains Study (KAPS) (1): a prospective cohort survey study (2014-2016) in which patients who consulted their general practitioner (GP), with one of the five most common musculoskeletal pain presentations (back, knee, shoulder, neck or multi-site pain), were invited to take part (14 general practices involved). Patients were sent an invitation letter and survey pack (containing the risk stratification tool) within two weeks of the initial GP consultation for their musculoskeletal pain. Return of the completed questionnaire included consent to participate in the cohort, which typically was received 3-6 weeks after the GP consultation. Follow-up questionnaires were mailed to participants at 2-months and 6-months after baseline. In total 1890 patients (465 with NLBP) participated with a 76% response at 2-months and a 79% response at 6-month follow-up.

ii) The STarT MSK Pilot Trial (STarT MSK-pilot) (2): a pragmatic, two-parallel arm, pilot cluster randomised controlled trial (RCT) in 8 general practices (2016-2017), that tested the feasibility of stratified care using the Keele STarT MSK Tool and matched treatment options, in first-line decision-making at the point-of-consultation. Four GP practices were assigned to offer usual care, with the remaining four offering stratified care based on the Keele STarT MSK Tool and matched treatment options. Patients were sent an invitation letter and survey pack (containing the draft risk stratification tool) within a week following their GP consultation for musculoskeletal pain. Return of the completed questionnaire signified consent to participate in the data collection, which was typically received 2-4 weeks after the GP consultation. In total, 524 patients took part (214 with NLBP), and 6-month follow-up was available in 91.8% of participants.

External validation data

iii) *STarT MSK Main Trial (STarT MSK-MT)* (3)*:* a two parallel arm cluster RCT (2018–2019) aiming to determine whether stratified care, involving use of the STarT MSK Tool and matched treatment options was more effective than usual care. The results showed no significant differences in pain outcomes between the arms of the trial. Data collection was identical to the STarT MSK-pilot methods.

Patients at general practices assigned to usual care had responses to the predictor items recorded only through self-reported questionnaire, typically received 2-4 weeks after the GP consultation, while those consulting at GP practices assigned to the stratified care intervention arm had their responses recorded both at consultation, by their GP, and through the self-reported questionnaires. A total of 1211 (586 with NLBP) patients took part with 88.5% follow-up at 6 months.

Appendix II: Detailed sample size calculations

Model development

The sample size was fixed due to the size of the available datasets. We compared the available number of participants (shown in figure 1 for each analysis) to sample size recommendations for developing prediction models with continuous (4) and binary outcomes (5).

Based on the anticipated inclusion of 11 pre-defined predictor parameters (one continuous predictor, modelled linearly, and 10 binary predictors), we required 311 participants for the development of models for continuous pain intensity (assumed R^2=0.22 (1), mean pain score 5.3 with standard deviation 2.2 (6)). The available data was sufficient to meet these recommendations for continuous outcomes at both time points.

For binary pain outcomes, model development required at least 824 participants (with 412 "moderate-high pain" events, assuming an outcome prevalence of 50%, 11 predictor parameters (as before), and a default Nagelkerke's R^2=0.15 (5)). For the binary work absence model, 1574 participants were required (with 244 work absence events, assuming an outcome prevalence of 15.5% (7), 16 predictor parameters (11 as before, plus four additional continuous predictors, modelled linearly, and one additional binary predictor) and a default Nagelkerke's R^2=0.15 (5)). The available data contained fewer than this recommended number of events for all binary outcomes.

External validation

The sample for external validation was again fixed to the size of the available external dataset. We compared the available number of participants to recommendations for the minimum sample size required to externally validate prediction models with continuous (8) and binary (8-10) outcomes.

Basing calculations on each model's performance on internal validation, the minimum sample size required to meet the Archer et al criteria (8) for the continuous pain models at both 2 and 6 months was 892 (assuming an R^2=0.39).

To meet the Collins et al recommendations (11) a minimum of 200 events (defined here as moderate-high pain) and non-events were required to externally validate each of the binary outcome prediction models.

To meet the Riley et al criteria (10) for external validation of the binary outcome models at 2 and 6 months, we required at least 1932 (1159 events) and 1946 (1071 events) participants respectively, driven by the criterion to precisely estimate to calibration slope. These calculations were based on requirements for precise estimation of O/E, c-slope, and c-statistic, and involved the following assumptions, taken from each model's performance on internal validation:

- 2-month moderate-high pain: outcome proportion of 60%, c-statistic of 0.84 and 0.81, linear predictor following a skew-normal distribution with a mean of -0.44, a variance of 2.20, a skewness parameter of −0.5, and a kurtosis parameter of 3.
- 6-month moderate-high pain: outcome proportion of 55%, c-statistic of 0.81, linear predictor following a skew-normal distribution with a mean of -0.45, a variance of 2.17, a skewness parameter of −0.5, and a kurtosis parameter of 3.

Appendix III: Extended statistical methods

Missing data

Multiple imputation by chained equations was used to account for missing data in both predictor and outcome measurements, under the assumption that data were missing at random (12, 13). Multiple imputation was performed separately for each dataset to allow for the clustering of individuals within that dataset. Preliminary checks for associations between missingness and predictor values were conducted to check for obvious violations of the missing at random assumption.

Several auxiliary variables were included in the imputation models, to increase precision and decrease bias in the prediction model estimates (14). which. These auxiliary variables included: self-rated health, intensity of least painful pain, EQ-5D-5L mobility domain, EQ-5D-5L anxiety/depression domain, co-morbidities (diabetes, breathing problems, heart problems, chronic fatigue, anxiety/depression and other), health literacy, and fear of pain-related movement. These variables were included in the imputation model only, and were not considered as predictors in the prognostic models.

The number of imputations generated was chosen to exceed the percentage of incomplete cases in the dataset (13). For model development, the maximum percentage of incomplete cases across the two datasets was used as the number of imputations for both datasets, to ensure they could easily be combined for analysis. The continuous outcome variables were included in the imputation models in their continuous forms to maximise the available information included in the imputation model. Outcome values were therefore imputed for individuals with missing outcome measurements as a part of the imputation process, however, participants who originally (prior to imputation) were missing data for an outcome were removed prior to any analyses related to that specific outcome (15).

Imputed values for all variables were checked through visual inspection of plots (continuous variables) and tables (categorical variables) to ensure values were realistic and consistent across imputed datasets. Results of analyses were pooled across imputations using Rubin's rules where appropriate (12).

Internal validation

Predictive performance of the developed models was assessed through calibration for the continuous outcome models, and through calibration and discrimination for the binary outcome models (16). Calibration was assessed using the calibration slope, calibration-in-the-large (CITL), and the ratio of Observed to Expected cases (O/E, for binary outcome models only). Discrimination was assessed through the C-statistic. The proportion of variance in the outcome explained by the predictors in each model was determined using the adjusted R^2 (or pseudo R^2 for binary outcomes, using Nagelkerke and Cox-Snell approaches).

Internal validation was conducted simultaneously for all models, using bootstrapping with 1,000 samples, sampling with replacement from the original data (17). The full modelling process was repeated within each bootstrap sample, including multiple imputation (12). The predictive performance of the model developed within each bootstrap sample was evaluated within the bootstrap sample itself, as well as in the original imputed data. Average optimism (difference in predictive performance between the bootstrap and original datasets) was subtracted from the apparent performance measures (the performance of the prediction model developed and evaluated in the original data) to provide optimism-adjusted estimates of predictive performance (18).

The optimism-adjusted calibration slope was also an estimate of the uniform shrinkage factor for each model. The regression coefficients were multiplied by the shrinkage factor to correct for overfitting (a consequence of having a low number of outcomes relative to predictors considered) (17, 19). After shrinkage, the intercept term was re-estimated for each model, to ensure predictions

were correct on average while maintaining the random-effects specified above. The models with shrunken coefficients and re-estimated intercepts are reported as the final prognostic models (18). Calibration plots were produced to show the performance of these shrunken models in the original data.

Appendix IV: Supplementary Tables and Figures

Table S1: candidate predictor definitions and coding

Item	Question phrasing	Possible values
Primary pain site	*When you recently visited your GP practice, which part of your body did you consult about?*	1 = back, 0 = neck
Pain intensity	*On average, how intense was your pain [where 0 is "no pain" and 10 is "pain as bad as it could be"]?*	0-10
Pain self-efficacy	*Do you often feel unsure about how to manage your pain condition?*	1 = yes, 0 = no
Pain impact	*Over the last two weeks, have you been bothered a lot by your pain?*	1 = yes, 0 = no
Walking short distances only	*Have you only been able to walk short distances because of your pain?*	1 = yes, 0 = no
Pain elsewhere	*Have you had troublesome joint or muscle pain in more than one part of your body?*	1 = yes, 0 = no
Thinking their condition will last a long time	*Do you think your condition will last a long time?*	1 = yes, 0 = no
Other important health problems	*Do you have other important health problems?*	1 = yes, 0 = no
Emotional well-being	*Has pain made you feel down or depressed in the last two weeks?*	1 = yes, 0 = no
Fear of pain-related movement	*Do you feel it is unsafe for a person with a condition like yours to be physically active?*	1 = yes, 0 = no
Pain duration	*Have you had your current pain problem for 6 months or more?*	1 = yes, 0 = no
Health literacy	*How often do you need to have someone help you when you read instructions on pamphlets, or other written material from your doctor or pharmacy [where 1 is "never" and 5 is "always"]?*	1-5
Work expectations	*In your estimation, what are the chances you will be working your normal duties in 3 months [where 0 is "no chance" and 10 is "very large chance"]?*	0-10
Pain interference	*During the past four weeks, how much did pain interfere with your normal work [where 1 is "not at all" and 5 is "extremely"]?*	1-5
Work satisfaction	*How satisfied are you with your employment? [where 1 is "very satisfied" and 4 is "severely dissatisfied"]*	1-4
Previous absence	*Have you taken time off work during the last 6 months because of your pain condition?*	1 = yes, 0 = no

Table S2: Internal validation performance of models for predicting pain

Time	Outcome	Measure	Apparent performance	Average optimism	Optimism adjusted
2 months	Pain score	Calibration slope	1 (0.894 to 1.106)	0.025 (0.024 to 0.026)	0.975
		CITL	0 (-0.193 to 0.193)	0.143 (0.139 to 0.147)	-0.143
		E/O	1 (1 to 1)	-0.031 (-0.032 to -0.031)	1.031
		R^2, median (IQR)	39.1 (38.8 to 39.5)		
	High pain	Calibration slope	0.999 (0.824 to 1.173)	0.069 (0.067 to 0.07)	0.930
		CITL	0.011 (-0.198 to 0.22)	1.241 (1.236 to 1.246)	-1.230
		E/O	0.996 (0.993 to 1)	-0.126 (-0.128 to -0.125)	1.123
		C-statistic	0.838 (0.805 to 0.871)	0.013 (0.012 to 0.013)	0.825
		Pseudo R^2, median (IQR)	43.2 (42.9 to 43.4)		
6 months	Pain score	Calibration slope	1 (0.895 to 1.105)	0.018 (0.017 to 0.018)	0.982
		CITL	0 (-0.193 to 0.193)	-0.547 (-0.551 to -0.544)	0.547
		E/O	1 (1 to 1)	0.133 (0.132 to 0.134)	0.867
		R^2, median (IQR)	39.1 (38.8 to 39.2)		0.37
	High pain	Calibration slope	0.994 (0.811 to 1.177)	0.056 (0.055 to 0.057)	0.938
		CITL	0.036 (-0.165 to 0.237)	0.329 (0.323 to 0.334)	-0.293
		E/O	0.986 (0.983 to 0.989)	0.014 (0.012 to 0.016)	0.972
		C-statistic	0.811 (0.775 to 0.847)	0.01 (0.01 to 0.01)	0.801
		Pseudo R^2, median (IQR)	37.5 (37.3 to 37.7)		0.33

Figure S1: Calibration plots for optimism-adjusted prediction models in model development data

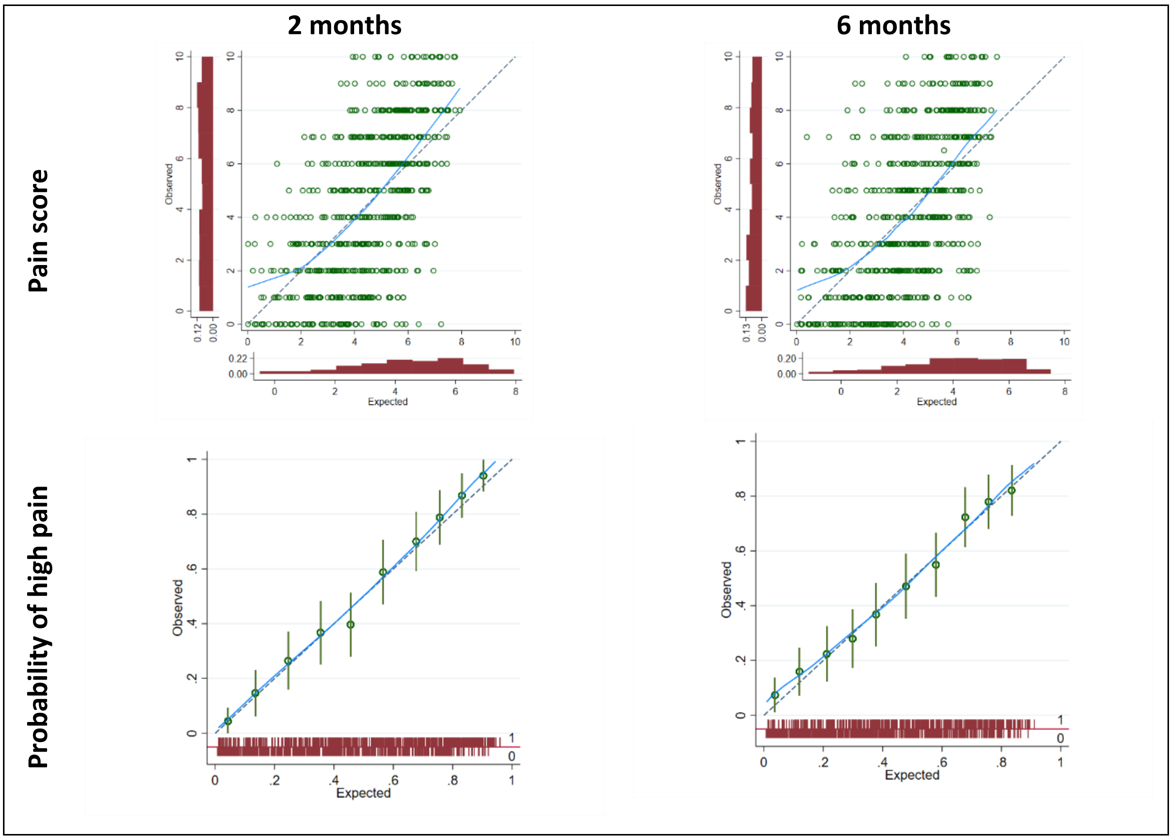

Appendix V: External validation performance in subgroups

As a representative example of the subgroup analyses across all models, and both external validation data types (point-of-consultation and 2-4 weeks after consultation) we present the external validation performance for the 6-month pain models across groups by age, sex, pain duration at presentation, and treatment arm in the STarT MSK main trial (treatment refers to matched treatment from STarT MSK tool score, and control refers to usual care).

Results are presented for predictions generated 2-4 weeks after consultation. Note that no sub-group had sufficient sample size for the external validation of a prediction model with either a continuous or binary outcome, as described in Appendix II, thus there is large amounts of uncertainty in some of these performance statistic estimates.

Age group

Age distribution, over full EV data and by outcome group

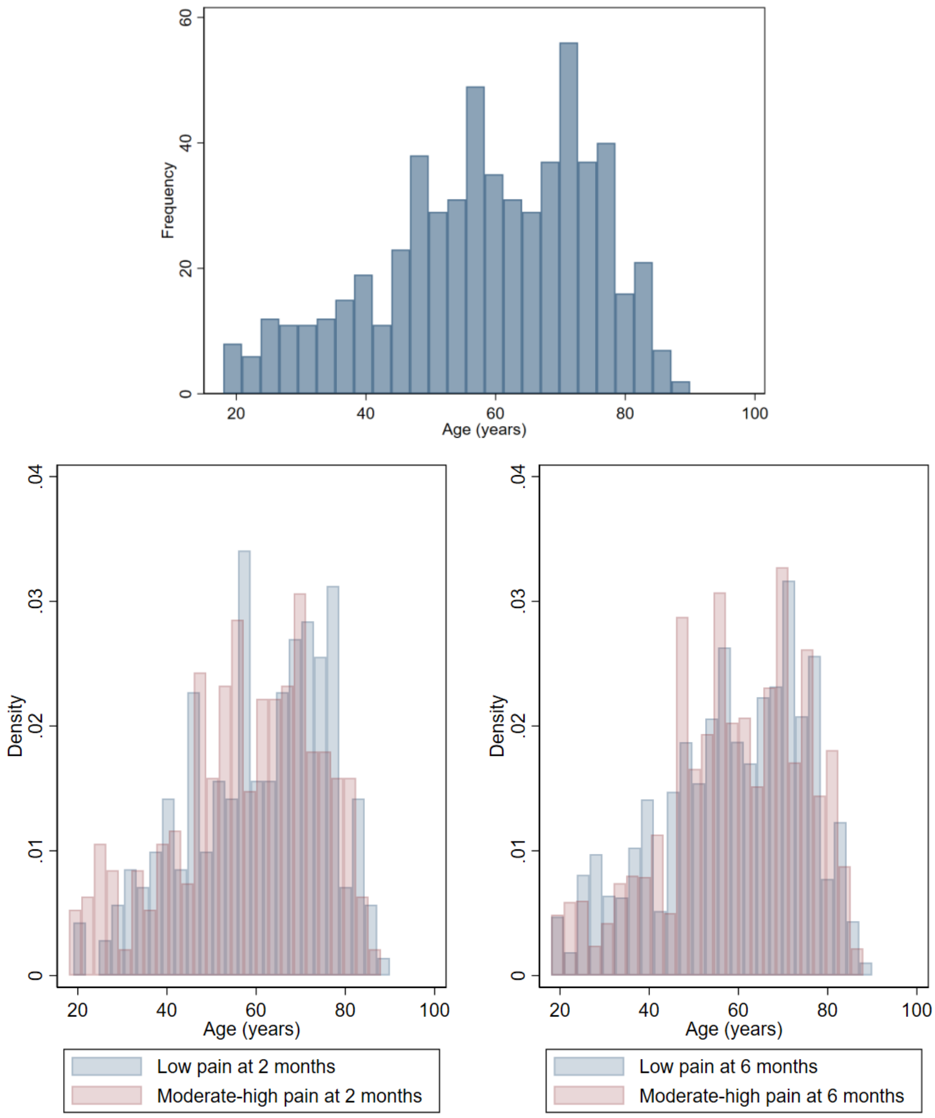

Model performance was assessed on external validation in the following age subgroups:

Age group	Number	Percentage
18-34	57	9.7%
35-49	109	18.6%
50-64	175	29.9%
65+	245	41.8%

Predictive performance statistics for models to predict pain at 6 months, using predictions generated in data collected 2-4 weeks after consultation:

Outcome	Measure	Age group			
		18-34	35-49	50-64	65+
6m pain score	Calibration slope	1.11 (0.5 to 1.71)	0.97 (0.72 to 1.21)	0.74 (0.46 to 1.03)	0.79 (0.6 to 0.99)
	CITL	-0.35 (-1.11 to 0.41)	-0.25 (-0.71 to 0.21)	-0.42 (-0.84 to 0.00)	-0.45 (-0.81 to -0.09)
6m high pain	Calibration slope	1.04 (0.09 to 1.99)	1.01 (0.55 to 1.48)	0.63 (0.3 to 0.97)	0.78 (0.49 to 1.06)
	CITL	-0.18 (-0.95 to 0.58)	-0.06 (-0.54 to 0.42)	-0.21 (-0.57 to 0.16)	-0.36 (-0.68 to -0.05)
	O/E	0.91 (0.86 to 0.96)	0.98 (0.92 to 1.04)	0.92 (0.87 to 0.98)	0.86 (0.81 to 0.91)
	C-statistic	0.76 (0.56 to 0.88)	0.8 (0.7 to 0.88)	0.68 (0.59 to 0.76)	0.73 (0.66 to 0.79)

Calibration of predictions for continuous pain score at 6 months:

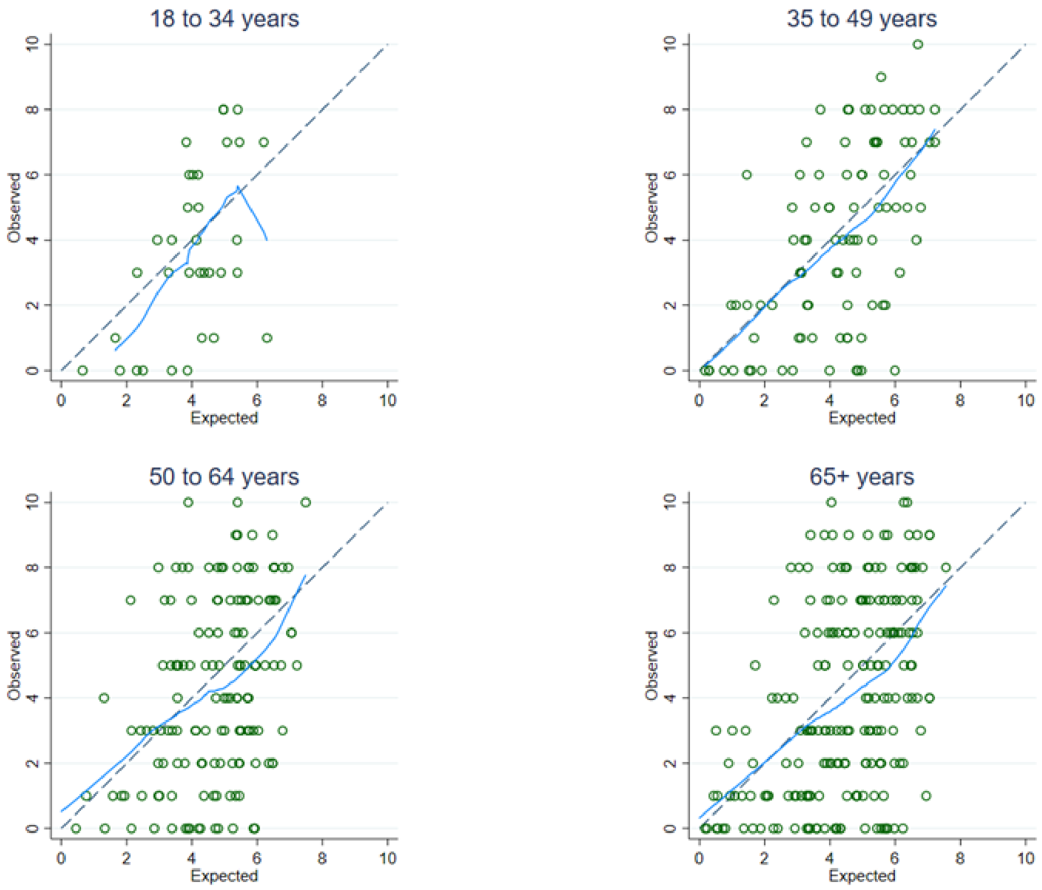

Calibration of predicted probabilities for moderate-high pain at 6 months:

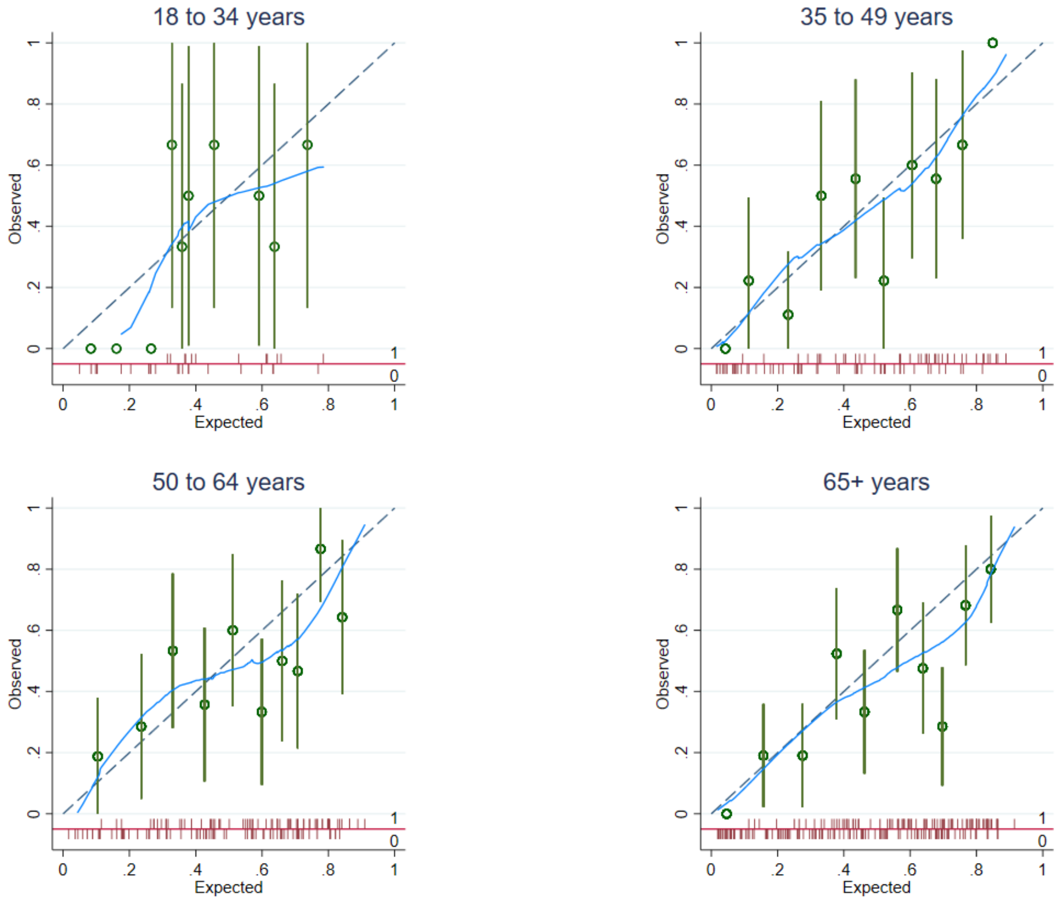

Sex

Predictive performance statistics for models to predict pain at 6 months, using predictions generated in data collected 2-4 weeks after consultation:

Outcome	Measure	Sex	
		Male	Female
6m pain score	Calibration slope	0.85 (0.66 to 1.05)	0.82 (0.64 to 1.00)
	CITL	-0.39 (-0.73 to -0.05)	-0.4 (-0.7 to -0.1)
6m high pain	Calibration slope	0.96 (0.64 to 1.29)	0.7 (0.46 to 0.93)
	CITL	-0.1 (-0.43 to 0.23)	-0.34 (-0.6 to -0.08)
	O/E	0.96 (0.9 to 1.02)	0.87 (0.82 to 0.93)
	C-statistic	0.77 (0.7 to 0.83)	0.7 (0.64 to 0.76)

Calibration of predictions for continuous pain score at 6 months:

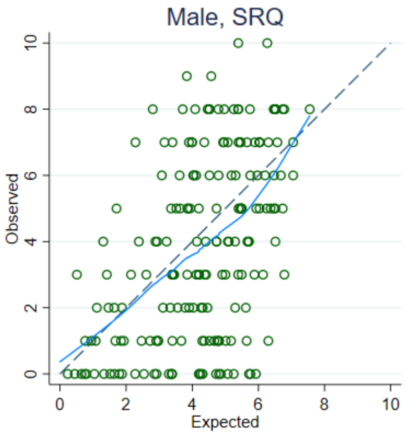

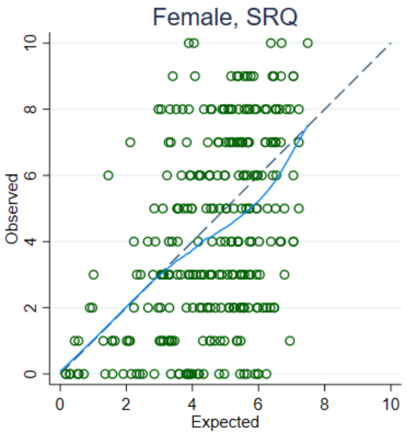

Calibration of predicted probabilities for moderate-high pain at 6 months:

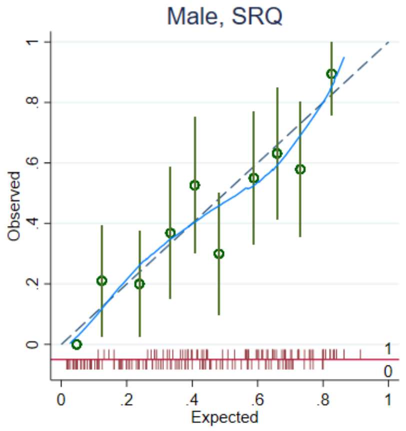

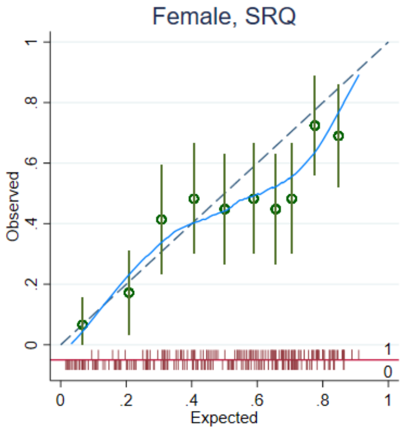

Trial arm

Information for both groups only available for predictor items measured via self-report questionnaire. “Intervention” implies matched treatment, “Control” implies usual care.

Predictive performance statistics for models to predict pain at 6 months, using predictions generated in data collected 2-4 weeks after consultation:

Outcome	Measure	Trial arm	
		Intervention	Control
6m pain score	Calibration slope	0.77 (0.56 to 0.99)	0.89 (0.72 to 1.06)
	CITL	-0.6 (-0.94 to -0.25)	-0.24 (-0.53 to 0.06)
6m high pain	Calibration slope	0.65 (0.38 to 0.93)	0.92 (0.65 to 1.18)
	CITL	-0.41 (-0.72 to -0.11)	-0.1 (-0.38 to 0.17)
	O/E	0.84 (0.79 to 0.89)	0.96 (0.9 to 1.02)
	C-statistic	0.68 (0.61 to 0.75)	0.77 (0.71 to 0.82)

Calibration of predictions for continuous pain score at 6 months:

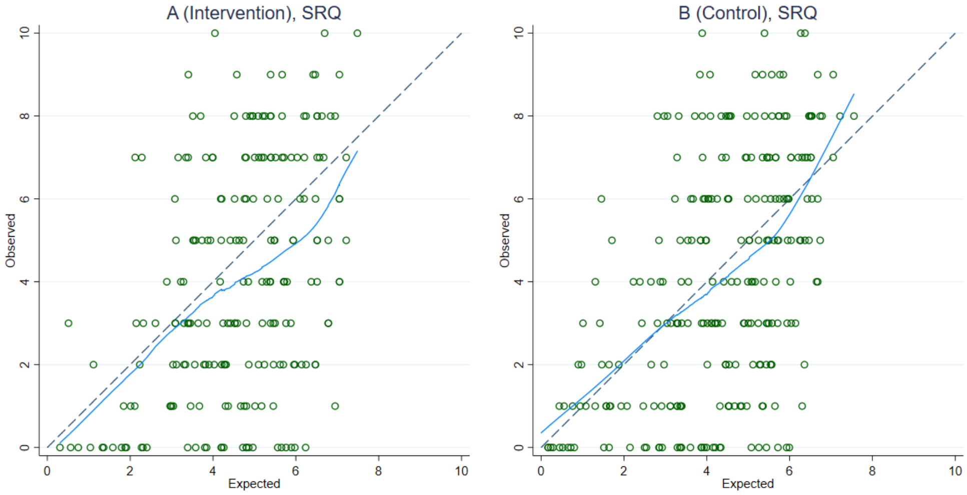

Calibration of predicted probabilities for moderate-high pain at 6 months:

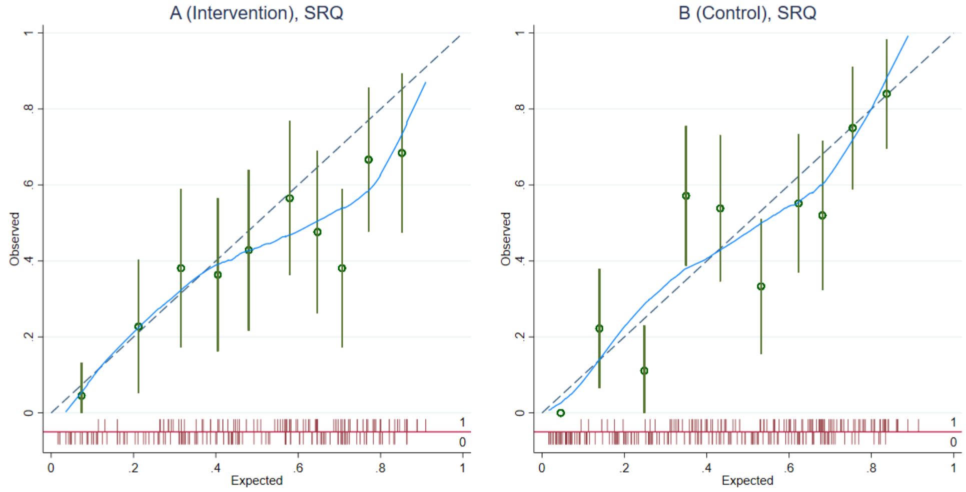

Pain duration

Predictive performance statistics for models to predict pain at 6 months, using predictions generated in data collected 2-4 weeks after consultation:

Outcome	Measure	Pain duration group			
		< 3 months	3-6 months	7-12 months	1 year+
6m pain score	Calibration slope	0.81 (0.56 to 1.06)	0.5 (0.2 to 0.81)	0.59 (0.1 to 1.08)	0.86 (0.65 to 1.06)
	CITL	-0.48 (-0.96 to -0.01)	-1.14 (-1.7 to -0.57)	-0.47 (-1.21 to 0.27)	-0.12 (-0.42 to 0.18)
6m high pain	Calibration slope	0.9 (0.48 to 1.32)	0.67 (0.16 to 1.17)	0.79 (0.21 to 1.36)	0.69 (0.42 to 0.95)
	CITL	-0.5 (-0.95 to -0.06)	-0.88 (-1.5 to -0.27)	-0.07 (-0.66 to 0.52)	-0.03 (-0.31 to 0.26)
	O/E	0.79 (0.75 to 0.84)	0.63 (0.6 to 0.66)	0.97 (0.91 to 1.03)	0.99 (0.93 to 1.05)
	C-statistic	0.77 (0.68 to 0.85)	0.71 (0.56 to 0.82)	0.71 (0.55 to 0.82)	0.69 (0.62 to 0.75)

Calibration of predictions for continuous pain score at 6 months:

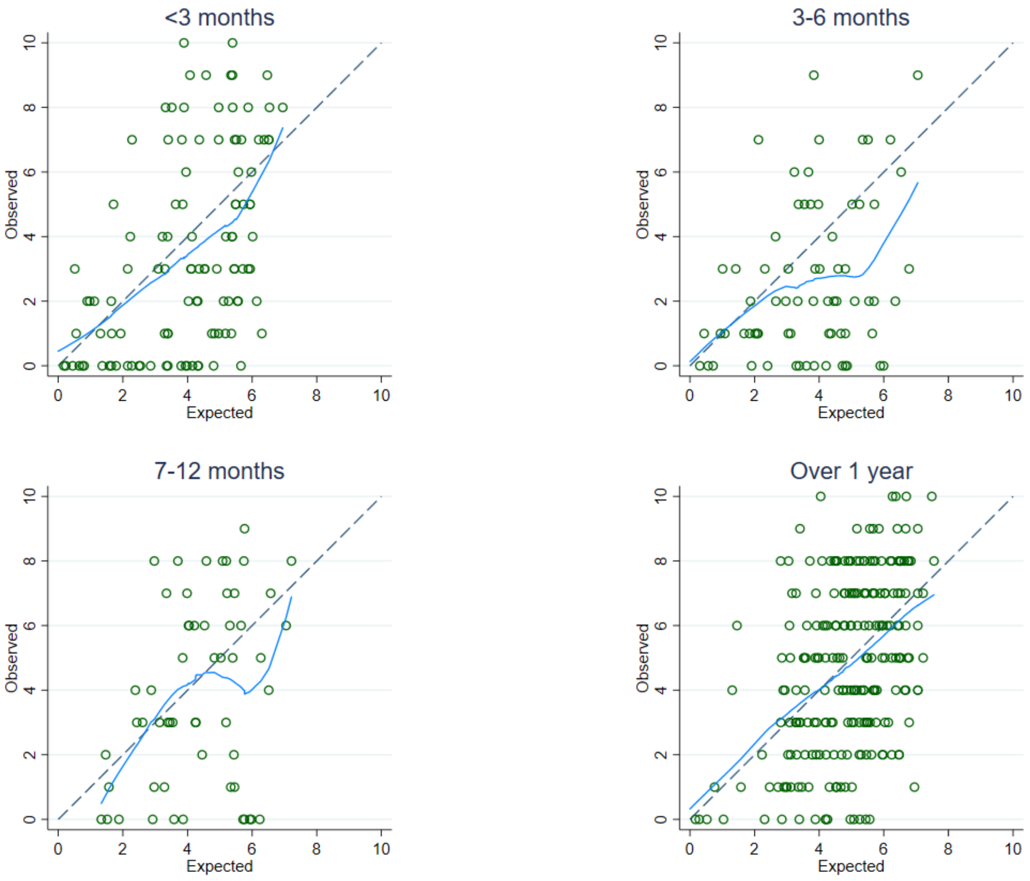

Calibration of predicted probabilities for moderate-high pain at 6 months:

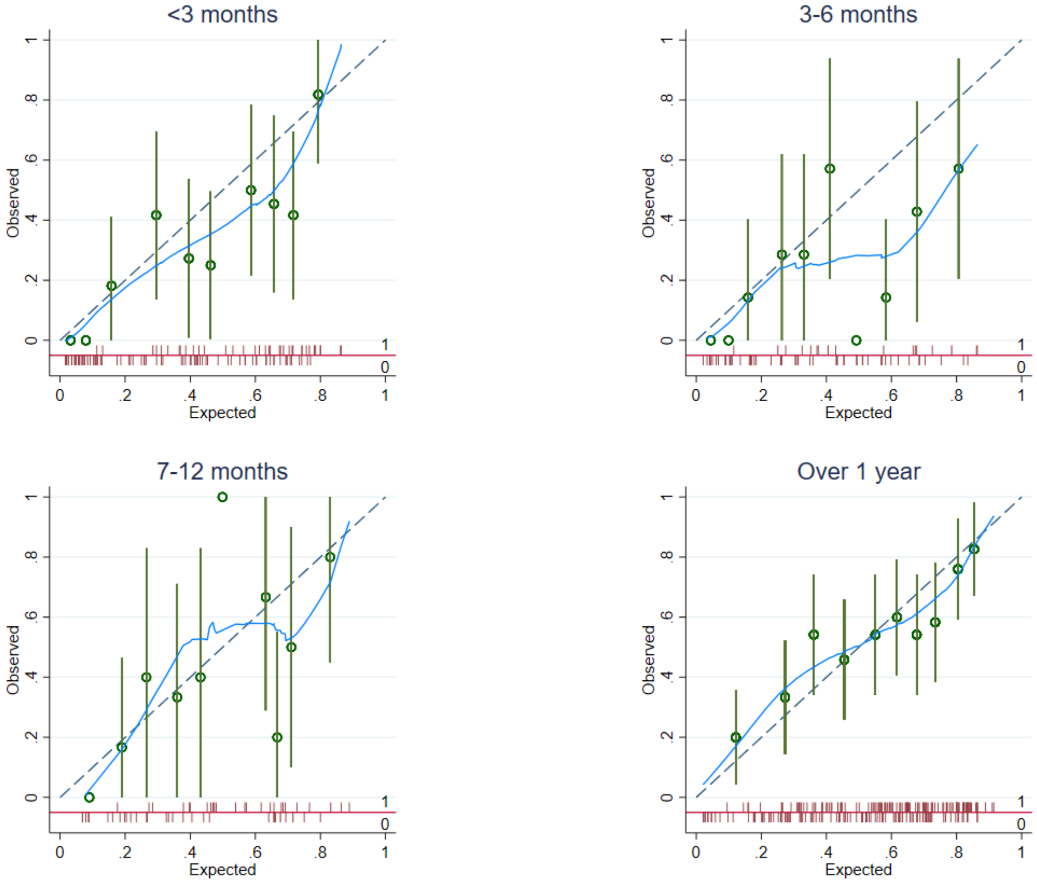

Appendix VI: Tripod checklist

Section/Topic			Checklist Item	Page
**Title and abstract**				
Title	1	D;V	Identify the study as developing and/or validating a multivariable prediction model, the target population, and the outcome to be predicted.	1
Abstract	2	D;V	Provide a summary of objectives, study design, setting, participants, sample size, predictors, outcome, statistical analysis, results, and conclusions.	3-4
**Introduction**				
Background and objectives	3a	D;V	Explain the medical context (including whether diagnostic or prognostic) and rationale for developing or validating the multivariable prediction model, including references to existing models.	5-6
	3b	D;V	Specify the objectives, including whether the study describes the development or validation of the model or both.	6
**Methods**				
Source of data	4a	D;V	Describe the study design or source of data (e.g., randomized trial, cohort, or registry data), separately for the development and validation data sets, if applicable.	7, AI
	4b	D;V	Specify the key study dates, including start of accrual; end of accrual; and, if applicable, end of follow-up.	7, AI
Participants	5a	D;V	Specify key elements of the study setting (e.g., primary care, secondary care, general population) including number and location of centres.	4, AI
	5b	D;V	Describe eligibility criteria for participants.	7
	5c	D;V	Give details of treatments received, if relevant.	NA
Outcome	6a	D;V	Clearly define the outcome that is predicted by the prediction model, including how and when assessed.	7
	6b	D;V	Report any actions to blind assessment of the outcome to be predicted.	NA
Predictors	7a	D;V	Clearly define all predictors used in developing or validating the multivariable prediction model, including how and when they were measured.	8/table S1
	7b	D;V	Report any actions to blind assessment of predictors for the outcome and other predictors.	NA
Sample size	8	D;V	Explain how the study size was arrived at.	8-9, AII
Missing data	9	D;V	Describe how missing data were handled (e.g., complete-case analysis, single imputation, multiple imputation) with details of any imputation method.	9, AIII
Statistical analysis methods	10a	D	Describe how predictors were handled in the analyses.	9-10, AIII
	10b	D	Specify type of model, all model-building procedures (including any predictor selection), and method for internal validation.	9-10
	10c	V	For validation, describe how the predictions were calculated.	10
	10d	D;V	Specify all measures used to assess model performance and, if relevant, to compare multiple models.	10
	10e	V	Describe any model updating (e.g., recalibration) arising from the validation, if done.	NA
Risk groups	11	D;V	Provide details on how risk groups were created, if done.	NA
Development vs. validation	12	V	For validation, identify any differences from the development data in setting, eligibility criteria, outcome, and predictors.	12-13
**Results**				
Participants	13a	D;V	Describe the flow of participants through the study, including the number of participants with and without the outcome and, if applicable, a summary of the follow-up time. A diagram may be helpful.	Fig 1
	13b	D;V	Describe the characteristics of the participants (basic demographics, clinical features, available predictors), including the number of participants with missing data for predictors and outcome.	Table 1
	13c	V	For validation, show a comparison with the development data of the distribution of important variables (demographics, predictors and outcome).	Table 1
Model development	14a	D	Specify the number of participants and outcome events in each analysis.	Fig 1
	14b	D	If done, report the unadjusted association between each candidate predictor and outcome.	NA
Model specification	15a	D	Present the full prediction model to allow predictions for individuals (i.e., all regression coefficients, and model intercept or baseline survival at a given time point).	Table 3
	15b	D	Explain how to the use the prediction model.	Box 1
Model performance	16	D;V	Report performance measures (with CIs) for the prediction model.	Table 4
Model-updating	17	V	If done, report the results from any model updating (i.e., model specification, model performance).	NA
**Discussion**				
Limitations	18	D;V	Discuss any limitations of the study (such as nonrepresentative sample, few events per predictor, missing data).	18-19
Interpretation	19a	V	For validation, discuss the results with reference to performance in the development data, and any other validation data.	16
	19b	D;V	Give an overall interpretation of the results, considering objectives, limitations, results from similar studies, and other relevant evidence.	16-20
Implications	20	D;V	Discuss the potential clinical use of the model and implications for future research.	19-20

Other information				
Supplementary information	21	D;V	Provide information about the availability of supplementary resources, such as study protocol, Web calculator, and data sets.	-
Funding	22	D;V	Give the source of funding and the role of the funders for the present study.	2

References

1. Dunn K, Campbell P, Lewis M, Hill J, van der Windt D, Afolabi E, et al. Refinement and validation of a tool for stratifying patients with musculoskeletal pain. European Journal of Pain. 2021.
2. Hill J, Garvin S, Chen Y, Cooper V, Wathall S, Saunders B, et al. Stratified primary care versus non-stratified care for musculoskeletal pain: findings from the STarT MSK feasibility and pilot cluster randomized controlled trial. BMC Family Practice. 2020;21(30).
3. Hill JC, Garvin S, Bromley K, Saunders B, Kigozi J, Cooper V, et al. Risk-based stratified primary care for common musculoskeletal pain presentations (STarT MSK): a cluster-randomised, controlled trial. The Lancet Rheumatology. 2022.
4. Riley RD, Snell KIE, Ensor J, Burke DL, Harrell FE, Jr., Moons KGM, et al. Minimum sample size for developing a multivariable prediction model: Part I - Continuous outcomes. Statistics in medicine. 2019;38(7):1262-75.
5. Riley RD, Snell KI, Ensor J, Burke DL, Harrell FE, Jr., Moons KG, et al. Minimum sample size for developing a multivariable prediction model: PART II - binary and time-to-event outcomes. Stat Med. 2019;38(7):1276-96.
6. Hill J, Whitehurst D, Lewis M, Bryan S, Dunn K, Foster N, et al. Comparison of stratified primary care management for low back pain with current best practice (STarT Back): a randomised controlled trial. Lancet 2011;378(9802):1560-71.
7. Wynne-Jones G, Cowen J, Jordan JL, Uthman O, Main CJ, Glozier N, et al. Absence from work and return to work in people with back pain: a systematic review and meta-analysis. Occupational and Environmental Medicine. 2014;71:448-56.
8. Archer L, Snell KI, Ensor J, Hudda MT, Collins GS, Riley RD. Minimum sample size for external validation of a clinical prediction model with a continuous outcome. Stat Med. 2020;40(1):133-46.
9. Snell KI, Archer L, Ensor J, Bonnett LJ, Debray TP, Phillips B, et al. External validation of clinical prediction models: simulation-based sample size calculations were more reliable than rules-of-thumb. J Clin Epidemiol. 2021;S0895-4356(21)00048-2.
10. Riley RD, Debray TPA, Collins GS, Archer L, Ensor J, van Smeden M, et al. Minimum sample size for external validation of a clinical prediction model with a binary outcome. Statistics in Medicine. 2021;40(19):4230-51.
11. Collins GS, Ogundimu EO, Altman DG. Sample size considerations for the external validation of a multivariable prognostic model: a resampling study. Stat Med. 2016;35(2):214-26.
12. Vergouwe Y, Royston P, Moons KGM, Altman DG. Development and validation of a prediction model with missing predictor data: a practical approach. Journal of Clinical Epidemiology. 2010;63(2):205-14.
13. White IR, Royston P, Wood AM. Multiple imputation using chained equations: Issues and guidance for practice. Statistics in Medicine. 2011;30(4):377-99.
14. Hardt J, Herke M, Leonhart R. Auxiliary variables in multiple imputation in regression with missing X: a warning against including too many in small sample research. BMC Med Res Methodol. 2012;12:184.
15. Kontopantelis E, White I, Sperrin M, et al. Outcome-sensitive multiple imputation: a simulation study. BMC Med Res Methodol. 2017;17(2).
16. Altman DG, Vergouwe Y, Royston P, Moons KGM. Prognosis and prognostic research: validating a prognostic model. BMJ (Clinical research ed). 2009;338:b605.
17. Steyerberg EW. Clinical prediction models: A practical approach to development, validation, and updating. New York, NY: Springer; 2009.
18. Moons KGM, Altman DG, Reitsma JB, et al. Transparent Reporting of a multivariable prediction model for Individual Prognosis Or Diagnosis (TRIPOD): Explanation and Elaboration. Annals of Internal Medicine. 2015;162(1):W1-W73.

19. Van Houwelingen J, Le Cessie S. Predictive value of statistical models. Stat Med. 1990;9(11).
